# Controlling the electro-optic response of a semiconducting perovskite coupled to a phonon-resonant cavity

**DOI:** 10.1038/s41377-023-01232-0

**Published:** 2023-07-26

**Authors:** Lucia Di Virgilio, Jaco J. Geuchies, Heejae Kim, Keno Krewer, Hai Wang, Maksim Grechko, Mischa Bonn

**Affiliations:** 1 Max Planck Institute for Polymer Research, Ackermannweg 10, 55128 Mainz, Germany; 2 Pohang University of Science and Technology, Department of Physics, 37673 Pohang, Korea

**Keywords:** Terahertz optics, Optoelectronic devices and components

## Abstract

Optical cavities, resonant with vibrational or electronic transitions of material within the cavity, enable control of light-matter interaction. Previous studies have reported cavity-induced modifications of chemical reactivity, fluorescence, phase behavior, and charge transport. Here, we explore the effect of resonant cavity-phonon coupling on the transient photoconductivity in a hybrid organic-inorganic perovskite. To this end, we measure the ultrafast photoconductivity response of perovskite in a tunable Fabry–Pérot terahertz cavity, designed to be transparent for optical excitation. The terahertz-cavity field-phonon interaction causes apparent Rabi splitting between the perovskite phonon mode and the cavity mode. We explore whether the cavity-phonon interaction affects the material’s electron-phonon interaction by determining the charge-carrier mobility through photoconductivity. Despite the apparent hybridization of cavity and phonon modes, we show that the perovskite properties in both ground (phonon response) and excited (photoconductive response) states remain unaffected by the tunable light-matter interaction. Yet the response of the integral perovskite-terahertz optical cavity system depends critically on the interaction strength of the cavity with the phonon: the transient terahertz response to optical excitation can be increased up to threefold by tuning the cavity-perovskite interaction strength. These results enable tunable switches and frequency-controlled induced transparency devices.

## Abstract (web summary)

We investigate the ultrafast optoelectronic response of semiconducting perovskite in an optically transparent terahertz cavity. Despite apparent polariton formation and strong electron-phonon coupling, the cavity-coupled material properties remain unchanged.

## Graphical abstract

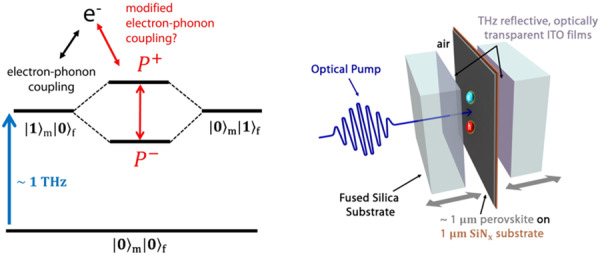

## Introduction

The selective interaction of coherent electromagnetic radiation with specific microscopic material motions (electronic and/or vibrational) can induce superconductivity^1^, optical phase transitions^2,3^, and influence molecular reaction pathways^4–6^. Exploiting the quantum nature of light has recently gained increasing attention as an alternative approach. In this method, the material behavior is modulated by tuning its interaction with the vacuum—rather than the coherent—state of the electromagnetic field^7,8^. Utilization of the vacuum state allows for avoiding limitations due to energy dissipation. The interaction between the quantum field and a two-level system causes the mixing of states |*i*〉_m_|*j*〉_f_, composed of material (|*i*〉_m_) and field (|*j*〉_f_) states, with different population quantum numbers *i* and *j*. The material-field interaction is typically weak and only slightly modulates material behavior (for example, the Lamb shift in hydrogen atoms)^9^. To enhance the coupling, the material can be placed inside a cavity that is tuned in resonance with a transition between the material’s two energy levels^7,10–12^. Strong mixing of field and material states using this method has been reported to produce polariton states for several systems (inorganic^13^, organic^14,15^ and hybrid perovskite^16,17^ materials) spanning from visible to terahertz (THz) frequencies^18–21^. This approach significantly affects the rate of chemical reactions^22–25^, the supramolecular self-assembly^26,27^, and the conductivity^28–33^.

**Fig. 1:**
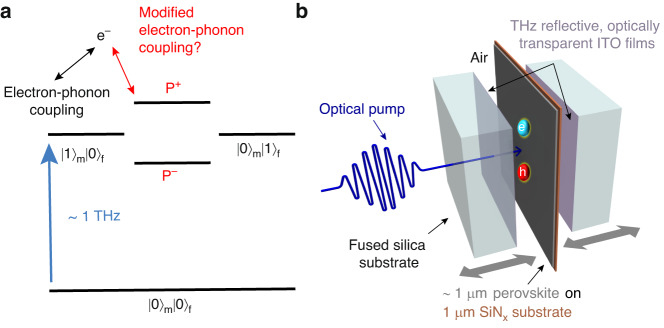
Electron-phonon coupling in an optically accessible, terahertz-tuneable cavity. **a** Schematic representation of the coupling of material and field states producing hybrid light-matter polariton states *P*^+^ and *P*^*−*^. We explore here whether the formation of phonon-polaritons can modify electron-phonon interactions. **b** Sketch of a perovskite thin film in an optically transparent Fabry–Pérot cavity, with a tunable cavity length of ~100’s of μm, so that its fundamental mode resonates at THz frequencies

Here, we investigate the resonant interaction between a terahertz (THz) field and organic-inorganic perovskite in a cavity and how it affects the electrical and optical properties of the system. We study methylammonium lead iodide (MAPI, (CH_3_NH_3_)PbI_3_) perovskite, which is composed of CH_3_NH_3_^+^ cations embedded inside a negatively charged inorganic PbI_3_^−^ sublattice^34^. Because of the local charges, i.e., the ionic nature of the crystal, the MAPI lattice is highly susceptible to external electric fields, and its phonon modes have high optical intensity^35,36^. On the one hand, the high intensity of the MAPI phonon modes provides an opportunity to couple them strongly with resonant THz modes of a cavity. On the other hand, phonons significantly influence charge mobility in MAPI due to strong electron-phonon interactions, giving rise to polaron formation and electron-phonon scattering^37^. The latter is the dominant mechanism inhibiting free charge motion in perovskite^37,38^. In harmonic systems, electron-phonon scattering occurs with dark longitudinal (LO) phonon modes^39^ that cannot couple with a transverse electromagnetic field. However, substantial anharmonicity in MAPI perovskite leads to the mixing of longitudinal with bright transverse (TO) phonon modes and even to actually localized character of these vibrations^35,40^. The latter is likely to enable a variety of scattering pathways for electrons by breaking momentum conservation rules. Thus, the coupling of bright phonons of MAPI perovskite with an electromagnetic cavity field can affect the density of states and the character of both TO and LO phonons and their interaction with charge carriers (Fig. 1a).

We tune the cavity in resonance with phonon modes of MAPI perovskite to enhance their interaction with the terahertz field. The cavity consists of two fused silica substrates, each with a deposited thin (190 nm) ITO layer (Fig. 1b). The ITO is transparent in the visible spectral range but is electrically conductive and, thus, reflects a major fraction of THz radiation. This design allows us to photoexcite charge carriers in the perovskite coupled with a THz cavity and probe the charge-carrier mobility using a THz pulse. It can provide an opportunity to control the conductivity properties of perovskites and the THz response of a perovskite-cavity system.

## Results

### Optical properties of the perovskite-cavity system

**Fig. 2:**
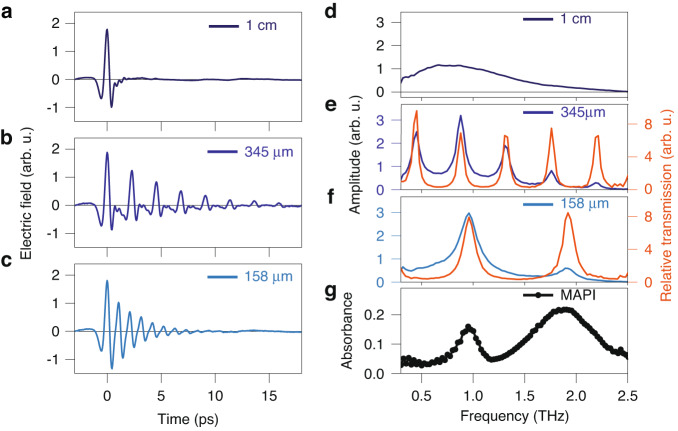
Optical properties of the THz cavity and the perovskite. **a** THz TDS of the empty cavity with a gap of *x*_*gap*_ = 1 cm; **b** *x*_*gap*_ = 345 μm; **c** *x*_*gap*_ = 158 μm. **d** Amplitude of the transmission obtained from the Fourier transform of the time trace in (**a**). **e**, **f** Frequency-domain transmission spectra for *x*_*gap*_ = 345 μm and 158 μm. Blue lines show the amplitudes of the transmission; orange lines show the transmission spectrum (amplitude normalized to the spectrum for *x*_*gap*_ = 1 cm). **g** Linear absorbance of methylammonium lead iodide (MAPI) perovskite outside the cavity in the THz frequency range

We characterize the performance of the cavity in the THz frequency range by measuring its transmission using terahertz time-domain spectroscopy (THz TDS)^41^. Figure 2a–f shows the time profile and spectrum of the THz pulse transmitted through the cavity with *x*_*gap*_ = 1 cm, 345 μm, and 158 μm spacing between the mirrors. For *x*_*gap*_ = 1 cm, the time delay between consecutive reflections of the THz pulse (≈66 ps) exceeds the time window of the THz TDS measurement (≈20 ps). Thus, this measurement characterizes the temporal shape *E*_*THz*_ (*t*) and spectrum ${E}_{{THz}}\left(\omega \right)$ of the pristine THz pulse (the optical response of the mirrors in this frequency range is shown in Supplementary Fig. S1). Upon reducing the cavity length, multiple replicas of the pulse reflecting between the two cavity mirrors appear with spacings of 2.3 ps (*x*_*gap*_ = 345 μm) and 1.05 ps (*x*_*gap*_ = 158 μm). The cavity has a quality factor of ~30 (see Supplementary Fig. S2), giving rise to a gradually decaying amplitude of the consecutive reflections (${E}_{{THz}}^{345}\left(t\right)$ and ${E}_{{THz}}^{158}\left(t\right)$, Fig. 2b, c). In the frequency domain, the spectra of the original pulse and its reflections interfere, producing characteristic spectra with intensity maxima (${{\rm{E}}}_{{\rm{THz}}}^{345}\left({\rm{\omega }}\right)$ and ${E}_{{THz}}^{158}\left(\omega \right)$, Fig. 2d–f). To derive the transmittance of the cavity, which depends only on the cavity parameters, we divide the intensity spectra ${\left|{E}_{{THz}}^{345}\left(\omega \right)\right|}^{2}$ and ${\left|{E}_{{THz}}^{158}\left(\omega \right)\right|}^{2}$ by the intensity spectrum ${\left|{{\rm{E}}}_{\text{THz}}\left({\rm{\omega }}\right)\right|}^{2}$. Depending on the cavity length, the transmittance spectra (Fig. 2d, f) contain a few to several peaks, which correspond to the longitudinal modes of the cavity separated by the free spectral range. The smaller the cavity length, the larger its free spectral range, and the sparser the cavity modes. Thus, adjusting *x*_*gap*_ allows tuning the cavity modes into resonance with specific optical transitions of a sample inside the cavity.

**Fig. 3:**
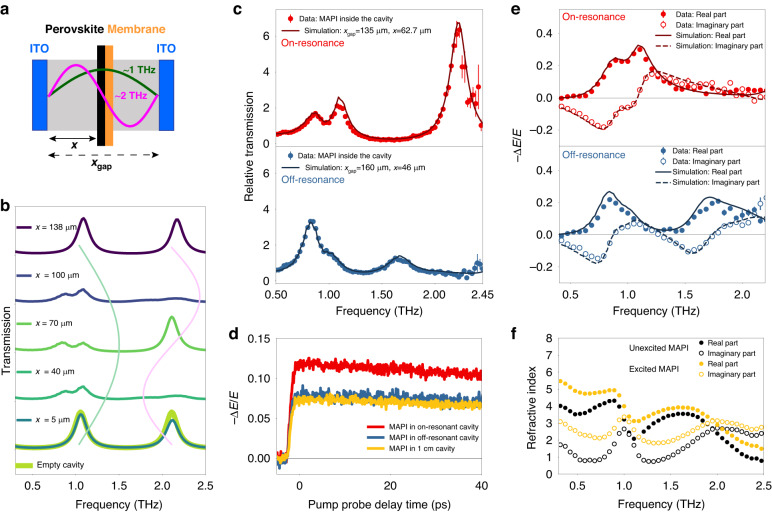
Frequency- and time-resolved spectra of the perovskite-cavity system. **a** Schematic of the perovskite-cavity system, with the cavity longitudinal *m* = 1 mode at ~1 THz. The field amplitudes of the *m* = 1 and 2 modes (with ~1 and ~2 THz frequency) appear in green and pink. **b** Simulations of the spectral transmission of the cavity-perovskite system as a function of the sample position in the cavity, with experimentally determined cavity and perovskite parameters. Depending on the position of the perovskite in the cavity, the local field strength (shaded lines) causes polariton formation at ~1 and ~2 THz. **c** Experimental (circles) and simulated transmission spectra, for *x*_*gap*_ = 135 μm and x = 63 μm (red line), *x*_*gap*_ = 160 μm and x = 46 μm (blue line). Estimated error bars are produced from four measurements. **d** Optical pump-THz probe signal of MAPI in the cavity with the same parameters as in (**c**), and also off-resonant cavity (*x*_*gap*_ = 1 cm, yellow line). Note the enhanced photoconductivity signal for the on-resonant case (red). **e** Experimental (circles) and simulated (thin lines) differential photoconductivity response for the same parameters as in *(***c**). Estimated error bars are produced from four measurements. **f** Refractive index of (photoexcited) MAPI outside the cavity, used to calculate the response in (**c**) and (**e**). All optical pump-THz probe measurements (inside as well as outside the cavity) are performed with an identical pump fluence of around 1 × 10^18^ photons m^−2^

We aim to couple these cavity modes with the optically active phonon vibrations of a MAPI perovskite sample. The perovskite has two intense modes in the frequency range of our THz pulse at ~1 THz and ~2 (Fig. 2g), which are assigned to the Pb-I-Pb bending vibration and the Pb–I bond stretch^42^. We insert a ~1-μm-thick polycrystalline sample supported on 1-μm-thick SiNx membrane^43^ inside the cavity (see Supplementary Fig. S3) and tune the first cavity mode (mode order m = 1) close to the resonance with the 1 THz phonon (Fig. 3a). With this cavity length, the second cavity mode (m = 2) is in resonance with the 2 THz perovskite phonon. However, the interaction of the electromagnetic field of the first and second cavity modes with the thin film sample is different because of the different spatial distributions of the field inside the cavity^44^. In the center of the cavity, the electric field is maximum for the m = 1 and zero for the m = 2 mode^44^. Thus, the interaction strength (the product of the electric field and the transition dipole moment) for different modes critically depends on the location of the sample inside the cavity. The perovskite-cavity system response shows large variability when the cavity length and/or position of the perovskite within the cavity are varied, as shown in simulations (detailed below) in Fig. 3b, and experimentally observed, as shown in Fig. 3c. Figure 3b shows that when the sample is near one of the mirrors (x = 5 and 138 μm), polariton formation is suppressed due to the low local field strengths, and the response of the systems closely resembles that of the empty cavity. In between these extrema, the local field strength of the 1 and 2 THz modes determines the field-matter interaction strength. The measurements in Fig. 3c confirm this behavior.

### Photoconductivity of the coupled perovskite-cavity system

MAPI is a semiconductor, and photoexcitation of its electrons from the valence to the conduction band induces conductivity. We probe conductivity by measuring the change in transmission of a THz pulse with and without optical excitation in an optical pump-THz probe (OPTP) experiment. To this end, we follow the standard approach and record the relative change −*∆E/E* of the THz electric field at the maximum of THz pulse^45–47^. Optical excitation of MAPI electrons by an ultrafast (≈50 fs, 400 nm central wavelength) laser pulse induces photoconductivity that decays on a time scale of >100 ps (Fig. 3d)^48^. After rapid cooling of the electrons within ~2 ps after photoexcitation, they reach a thermodynamic quasi-equilibrium state^49^ with a conductivity almost constant within our 40 ps time window. For identical excitation fluences (for details, see Supplementary Fig. S4), we observe indistinguishable charge-carrier dynamics for the MAPI sample without a cavity and at different locations inside the cavity (Fig. 3d). Remarkably, the signal at the peak intensity of THz pulse increases by a factor of ≈1.5 when the 1 THz mode of the cavity and perovskite phonon mode are resonant, as opposed to effectively non- (1 cm cavity) and off-resonant cavities. Under the resonant condition, Rabi splitting of 0.25 THz is slightly larger than 0.20 THz and 0.15 THz widths of the MAPI phonon and cavity modes, respectively. With these parameters, the perovskite-cavity system is in the intermediate (frequently also called strong^31,50–52^) coupling regime^53,54^. It is tempting to assign this increase in transient THz signal to enhanced charge mobility in perovskite, produced by the coupling between the electromagnetic field and the phonon mode. To test this hypothesis, we measure the photoconductivity spectrum 30 ps after photoexcitation when the sample reaches a quasi-steady state. The spectra look drastically different for the two perovskite-cavity configurations (Fig. 3e) across the entire frequency range of the THz pulse. Apparently, their complicated lineshapes show non-uniform changes in amplitude and phase of the THz pulse caused by photoexcitation. It is challenging to comprehend these results intuitively. Therefore, we perform classical electrodynamics calculations using the transmission matrix (T-matrix) formalism^55^ in order to understand their physical origin.

### Classical electrodynamics simulations of the (photoexcited) cavity-perovskite system

Our system, the cavity in resonance with the 1 THz phonon mode of MAPI, is composed of two reflecting ITO surfaces (refractive index independently determined, shown in Supplementary Fig. S1) separated by *x*_*gap*_ *=* 142 μm, a 1-μm-thick perovskite sample with the experimentally determined refractive index shown in Fig. 3f (details in Supplementary Note 3), and a 1-μm-thick SiNx substrate with a dispersionless refractive index of 2.8^56^. The calculated spectrum of the transmitted intensity (square of the electric field) is shown in Fig. 3b for various *x*. For *x* = 70 μm, the sample is near the node of the m = 2 cavity mode and interacts weakly with its field. Thus, the transmission spectrum around 2 THz resembles that of an empty cavity (Fig. 3b). In contrast, interaction with the m = 1 mode is strong, substantially modulating the transmission spectrum around 1 THz and splitting it into two peaks. This modulation results from the joint action of the imaginary (*k*(*ω*)) and real (*n*(*ω*)) parts of the sample refractive index. The former causes attenuation of the electromagnetic wave, and the latter changes the interference of the light propagating inside the cavity by changing the optical path length $n\left(\omega \right){x}_{{gap}}$^57^. For *x* = 40 μm and 100 μm, the sample interacts substantially with both cavity modes. At the extremes (*x* = 5 μm and 138 μm), interaction is weak due to the low electric field. These results show that interaction between field and a thin film sample is highly sensitive to the details of the configuration of the sample-cavity system, i.e., *x*_*gap*_, *x* and the sample properties.

Using this model of the system, we reproduce the experimental data by fine-tuning *x*_*gap*_ and *x*. The calculation results shown in Fig. 3c are in excellent agreement with the experiment. These calculations demonstrate that independent of the cavity length and sample location, the THz refractive index of the perovskite measured outside the cavity (see details in Supplementary Note 3 and Supplementary Fig. S5) is sufficient to reproduce the transmission spectra of the perovskite-cavity system. However, we observe that the entire system—i.e., the composite of the MAPI and the cavity—after photoexcitation changes its spectral response drastically as a function of both the cavity length and the position of the material, enabled by the tunable light-matter interaction.

To test if these spectra can likewise be explained by classical electrodynamics, we measure the refractive index of MAPI in free space (see Supplementary Fig. S6), at the same 30 ps time delay after photoexcitation, under identical excitation conditions (Fig. 3f). Note that presence of the cavity cannot cause interference effects for the pump pulse because the ITO coating is transparent at this wavelength. The thin lines in Fig. 3e show the photoconductivity spectra for the two configurations of the perovskite-cavity system calculated using T-matrix formalism and refractive index of ground-state and excited MAPI measured outside the cavity. This parameter-free description captures the data very well.

This excellent agreement demonstrates that classical electrodynamics can fully account for the complex, non-intuitive change of the perovskite transmission induced by its photoexcitation inside the cavity. In this model, the physical properties of MAPI do not change inside the cavity. Instead, the spectral lineshapes and photoconductivity dynamics are generated by wave absorption and interference, determined in a non-trivial manner by the cavity properties and the perovskite extinction coefficient and refractive index—different in the ground and excited states^57–59^. The change of the field modulation at the maximum of THz pulse in Fig. 3d is the result of complex interference of spectral components shown in Fig. 3e, with their different intensities and phases for different perovskite-cavity configurations.

### On-demand control of transient THz electric field

**Fig. 4:**
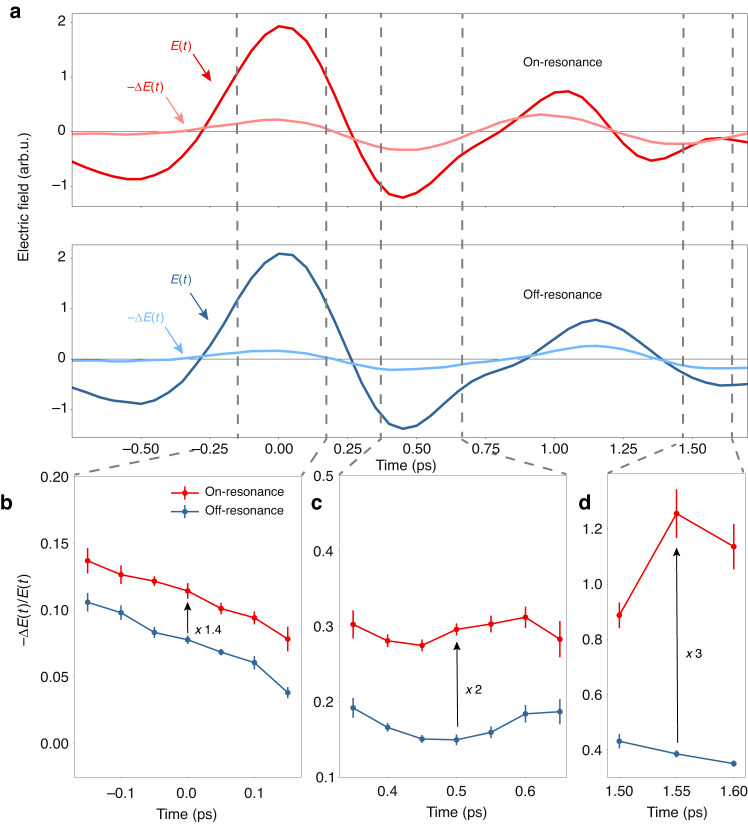
Cavity-tunable photoconductivity enhancement of the perovskite-cavity system. **a** Lines without symbols: Time trace of the pulsed THz field *E*(*t*) transmitted through the filled cavity on-resonance (red, *x*_*gap*_ = 135 μm and x = 62.7 μm) and off-resonance (blue, *x*_*gap*_ = 160 μm and x = 46 μm). Lines with dots: Relative change in the THz time profile $(-\Delta E(t)/E(t))$ for both configurations. **b**–**d** Zoom-in of the data around 0, 0.5, and 1.5 ps highlighted in panel (**a**), showing the relative signal enhancement for the resonant cavity

Even though the perovskite properties, both in the ground and excited states, do not change significantly inside the terahertz cavity, the response of the entire perovskite-cavity system shows substantial variations. The results in Fig. 3d demonstrate that the system enables tunable control of THz field modulation. These data represent the change of the maximum field of the THz pulse, as discussed above. Yet, modulation is not restricted to the maximum only. We now closely examine the data in the time domain, i. e. before the Fourier transformation that produced the spectra in Fig. 3e. For the two system configurations, Fig. 4a shows the electric field E(t) of the THz pulse and its photoinduced change $-\Delta E(t)$ measured 30 ps after sample excitation. The modulation depth of the THz field $-\Delta E(t)/E(t)$ shown in Fig. 4b–d for different time intervals varies substantially across the time profile of the THz pulse, as well as with cavity parameters. Before the maximum of the THz pulse, modulation of its field is identically weak for the on- and off-resonant perovskite-cavity configurations. Around the pulse maximum, the field changes by ~5–10% in the on-resonant perovskite-cavity system (Fig. 4b). At a later time of about 0.5 ps, modulation increases up to 20% (Fig. 4c) and reaches 40% at about 1.5 ps after the pulse maximum (Fig. 4d). Tuning the cavity into resonance with the 1 THz perovskite mode increases the modulation 1.5, 2 and 3-fold within duration of THz pulse, as shown in Fig. 4b–d. Such on-demand adjustability of ultrafast THz field modulation can benefit photonic integrated devices^60^ and optical communications modulation^61^.

## Discussion

In summary, we report the observation and simulation of the optoelectronic response of a semiconducting perovskite in an optically transparent Fabry–Pérot THz cavity using optical pump-terahertz probe spectroscopy. We reveal a tunable and on-demand control of the THz field modulation through the photoexcited perovskite-cavity system by tuning the cavity-material resonance. Our calculations demonstrate that, despite the apparent formation of polaritons between the cavity and perovskite modes, the material properties of the MAPI in the cavity remain unchanged. However, we observe that the entire MAPI-cavity system changes its spectral response as a function of the cavity length and the position of the material inside the cavity due to a tunable interaction between the refractive index of the material and the cavity resonances. The measured spectra of both unexcited and photoexcited MAPI inside the cavity can be very well described using classical electrodynamics. This work elucidates the role of cavity resonances in the presence of photoexcited media and opens the possibility of using a transparent THz optical cavity to shape the transmission of THz radiation and enhance on-demand THz field modulation through photoexcited semiconductor-cavity systems.

## Materials and methods

### Fabrication of THz cavity

To investigate the coupling between MAPI and cavity resonances, we designed and fabricated a tunable optically transparent THz cavity. The geometry of our system is schematically shown in Fig. 1a. The cavity is composed of two ITO films prepared by sputtering around 190 nm ITO layers on 2 mm-thick fused silica purchased from PI-KEM. The two ITO layers are reflective for THz radiation (ITO mirrors). They are mounted on two mirror mounts that allow fine alignment of the cavity. Both ITO mirrors are installed on two translation stages that allow varying the cavity length continuously and symmetrically around the THz focus (see Supplementary Fig. S3). The lateral size of the thin film MAPI (10 × 10 mm) is bigger than the size of mirrors (7 × 7 mm), as schematically shown in Fig. 1a.

### Perovskite film preparation

Methylammonium lead iodide is synthesized following the reaction:

$$\begin{array}{l}{\rm{Pb}}{({{\rm{CH}}}_{3}{\rm{COO}})}_{2}+3\,{{\rm{CH}}}_{3}{{\rm{NH}}}_{3}{\rm{I}}\to ({{\rm{CH}}}_{3}{{\rm{NH}}}_{3}){{\rm{PbI}}}_{3}\\+2\,{{\rm{CH}}}_{3}{{\rm{NH}}}_{3}{{\rm{CH}}}_{3}{\rm{COO}}\end{array}$$

The formed methylammonium acetate decomposes rapidly into methylamine (CH_3_NH_2_) and acetic acid (CH_3_COOH), which sublimate from the formed methylammonium lead iodide film upon annealing^62,63^. The thin MAPI film was prepared by spin-coating a solution composed of Methylammonium Iodide and Pb acetate in Dimethylformamide on a thin SiNx membrane. Methylammonium iodide (99.9% w/w, MAI) was purchased from Greatcell Energy. Lead acetate (98% w/w, Pb(Ac)_2_) was purchased from Tokyo Chemical Industry. Anhydrous dimethylformamide (>99.8% v/v, DMF) was purchased from Sigma Aldrich. SiNx (1 μm membrane thickness purchased from Norcada) membranes were subjected to UV-Ozone treatment (FHR UVOH 150 LAB, 250 W) for 20 min with an oxygen feeding rate of 1L min^−^^1^ right before spin-coating. The substrates were positioned in the center of a microscope slide and held in place with four pieces of electrical tape. Film preparation was carried out in a nitrogen-purged glovebox. The precursor solution consists of 477 mg (3 mmol) MAI and 325.3 mg of Pb(Ac)_2_ (1 mmol) in 1 mL DMF. The film preparation was done by depositing 50 μL of the precursor solution onto the SiNx membrane and spin-coating it at 3000 rpm (ramp ± 1000 rpm s^−1^) for 1 min. Afterward, the film was left to dry at room temperature for 5 min and subsequently annealed for 10 min on a 100 °C hotplate. The perovskite-coated membranes were mounted in custom-made holders and stored in the glovebox prior to their use (see more details about MAPI characterization in Supplementary Fig. S7).

### Optical pump THz probe spectroscopy

We use an amplified Ti:sapphire laser producing pulses with 800 nm central wavelength and ~50 fs pulse duration at 1 kHz repetition rate. The THz field is generated by optical rectification in a ZnTe crystal (thickness 1 mm, <110> orientation). The THz detection is based on the electro-optic effect in a second ZnTe crystal with 1 mm thickness. We vary the time delay between the THz field and the 800 nm sampling beam with a motorized delay stage (M-605.2DD purchased from Physik Instrument (PI)). More details of the THz setup are provided in ref. ^64^. The time delay between the optical pump and THz probe pulses is controlled by a second motorized delay stage (M-521.DD, Physik Instrument (PI)). The time step in OPTP measurements is 0.1 ps. The pump pulse has a 400 nm central wavelength and is produced by the second harmonic generation of an 800 nm femtosecond pulse in a beta barium borate crystal (BBO). We performed all measurements with incident photon density 1 × 10^18^ photons m^−2^. All frequency-resolved spectra are collected at a fixed pump-probe time delay (30 ps after photoexcitation) by scanning the sampling line^65^.

## Supplementary information

Supplementary Information for Controlling the electro-optic response of a semiconducting perovskite coupled to a phonon-resonant cavity

## Data Availability

The data that support the findings of this study are available from the corresponding authors upon reasonable request.
