# Supplementary material for: Controlling the electro-optic response of a semiconducting perovskite coupled to a phonon-resonant cavity
Source: Light Sci Appl. 2023 Jul 26;12:183. doi: 10.1038/s41377-023-01232-0 (PMC10368682; doi:10.1038/s41377-023-01232-0)
Supplement: Supplementary file 1 — Supplementary Information for Controlling the electro-optic response of a semiconducting perovskite coupled to a phonon-resonant cavity [file 41377_2023_1232_MOESM1_ESM.docx]

**Supplementary Information for**

Controlling the electro-optic response of a semiconducting perovskite coupled to a phonon-resonant cavity

Lucia Di Virgilio^1^, Jaco J. Geuchies^1^, Heejae Kim^1, 2^, Keno Krewer^1^, Hai Wang^1^, Maksim Grechko^1*^, Mischa Bonn^1*^

^1^ Max Planck Institute for Polymer Research, Ackermannweg 10, 55128 Mainz, Germany.

^2^ Pohang University of Science and Technology, Department of Physics, 37673 Pohang, Korea.

*Correspondence to: grechko@mpip-mainz.mpg.de, bonn@mpip-mainz.mpg.de

**Supplementary Note 1**

**Terahertz Time Domain Spectroscopy (THz-TDS) of ITO**

We measure the THz electric field transmitted through the fused silica substrate ($E_{Reference}$) and the ITO deposited on fused silica ($E_{Sample}$) as a function of time. To obtain conductivity spectrum $\sigma\left( \nu\right)$ of ITO (Fig. S1a) we perform Fourier transformation of each THz pulse and use an analytical thin film approximation (Tinkham approximation^1^) as follows:

$\sigma\left( \nu\right)=\left( -\frac{{\epsilon_{0}c (n}_{sub}+n_{air})}{L}\left( e^{\frac{2\pi i{(n}_{sub}-n_{air})\nu d}{c}}\frac{E_{Sample}\left( \nu\right)}{E_{Reference}\left( \nu\right)}-1 \right) \right)$.

$n_{sub}=1.96$ ^2^ is the refractive index of fused silica, $n_{air}=1$ the refractive index of the air,$\epsilon_{0}$ is the vacuum permittivity, c the speed of light in vacuum and $L$ the thickness of the thin ITO layer, $d$ is the difference of thickness between bare fused silica substrate and substrate coated with ITO.

We obtain the thickness of each substrate by measuring the time delay between the transmitted THz pulse and its reflected replica and using the refractive index $n_{sub}$ of silica^3^. The real part of the derived conductivity ($\sigma_{1}$) is around ${10}^{5}$ S m^-1^ (Fig. S1a). We use this value (and neglect imaginary part of $\sigma$) to calculate the ITO dielectric function $\varepsilon\left( \nu\right)$ given by:

$$\varepsilon\left( \nu\right)=\varepsilon_{1}\left( \nu\right)+\varepsilon_{2}\left( \nu\right)=1+\frac{i\sigma_{1}}{2\pi\nu\epsilon_{0}}$$

From this dielectric function we calculate the real ($n$) and imaginary ($k$) parts of the complex-valued refractive index (Fig. S1b):

$$n\left( \nu\right)=\left( \frac{1}{2}\left( \left( {\varepsilon_{1}\left( \nu\right)}^{2}+{\varepsilon_{2}\left( \nu\right)}^{2} \right)^{\frac{1}{2}}+\varepsilon_{1}\left( \nu\right) \right) \right)^{\frac{1}{2}}$$

$$k\left( \nu\right)=\left( \frac{1}{2}\left( {({\varepsilon_{1}\left( \nu\right)}^{2}+{\varepsilon_{2}\left( \nu\right)}^{2})}^{\frac{1}{2}}-\varepsilon_{1}\left( \nu\right) \right) \right)^{\frac{1}{2}}$$

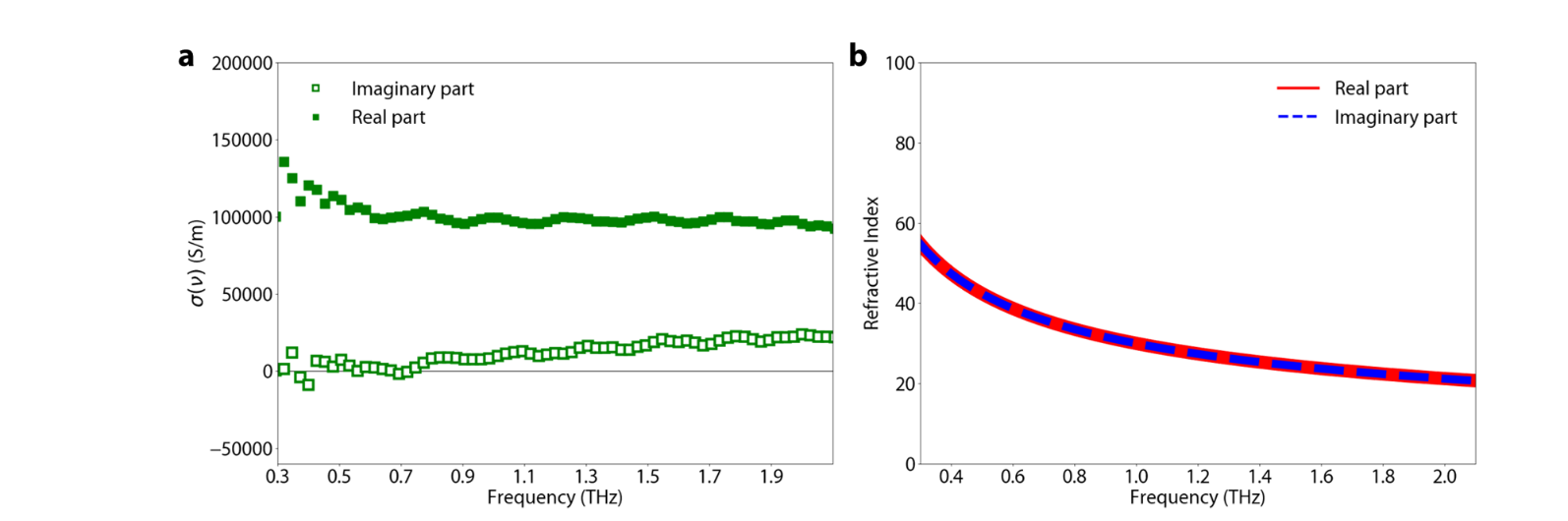


**Figure S1: Time Domain Spectroscopy (TDS) measurements of ITO. (a)** Complex conductivity of ITO from TDS measurement. **(b)** Complex refractive index of ITO, retrieved from the conductivity measurement in (a).


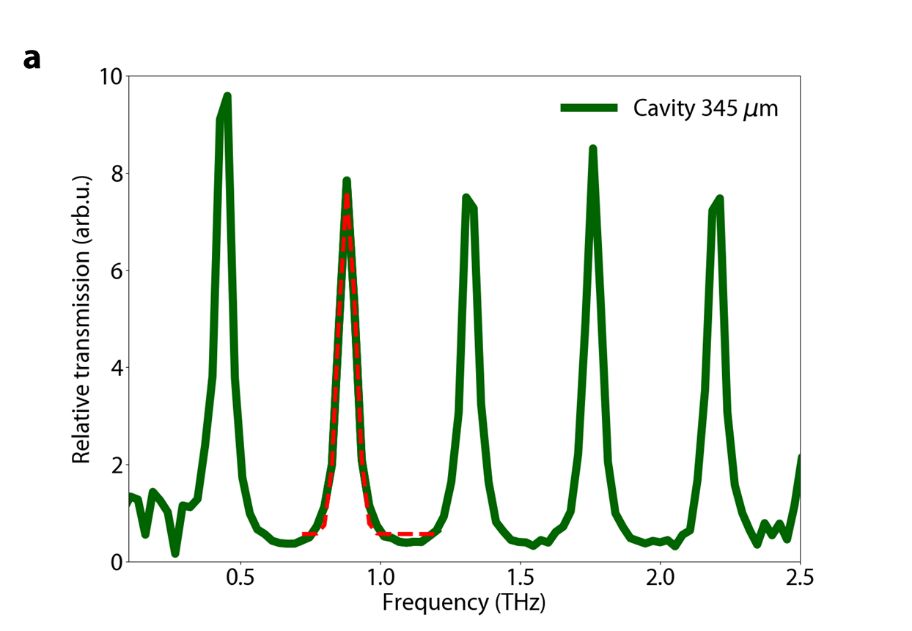


**Figure S2: Q-factor calculation.** The data from Fig. 2f of the manuscript (green) plotted together with fit to a Gaussian function (red). FWHM of the fitted trace is $0.0299\pm0.0009 \text{THz}$. We estimate the Q-factor to be about 30 by calculating $\frac{\nu_{r}}{FWHM}$, where with $\nu_{r}=0.8809\pm0.0008 THz$ is the resonance frequency.


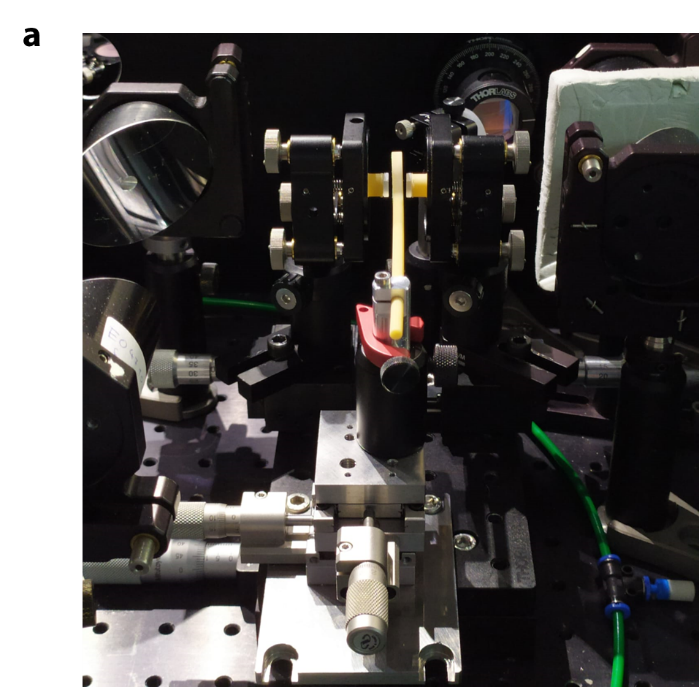


**Figure S3: THz cavity geometry.** Photograph of the cavity with the sample. The mirrors are installed on mirror mounts to allow fine adjustments of cavity alignment. The alignment is performed by using a diode laser and monitoring the interference in the beam reflected from the cavity. Both the mirrors and the sample are mounted on manual linear translation stages to tune their positions.

**Supplementary Note 2**

**Optical Pump THz probe (OPTP) spectroscopy**

In OPTP measurements MAPI is excited by pump pulse with 3.1 eV photon energy. The pump pulse is produced by second harmonic generation of femtosecond pulse (800 nm central wavelength) in a BBO crystal. The conductivity of the excited charge carriers is then probed by measuring the change in transmission of THz field through the sample. The time delay between optical pump and THz probe pulses is adjusted by using a motorized linear translation stage. All measurements of MAPI are performed with around $1\times{10}^{18}$ photons m^-2^ incident photon density.

OPTP measurements of the SiNx membrane and the empty cavity show negligible signal (Figs. S4c, d).


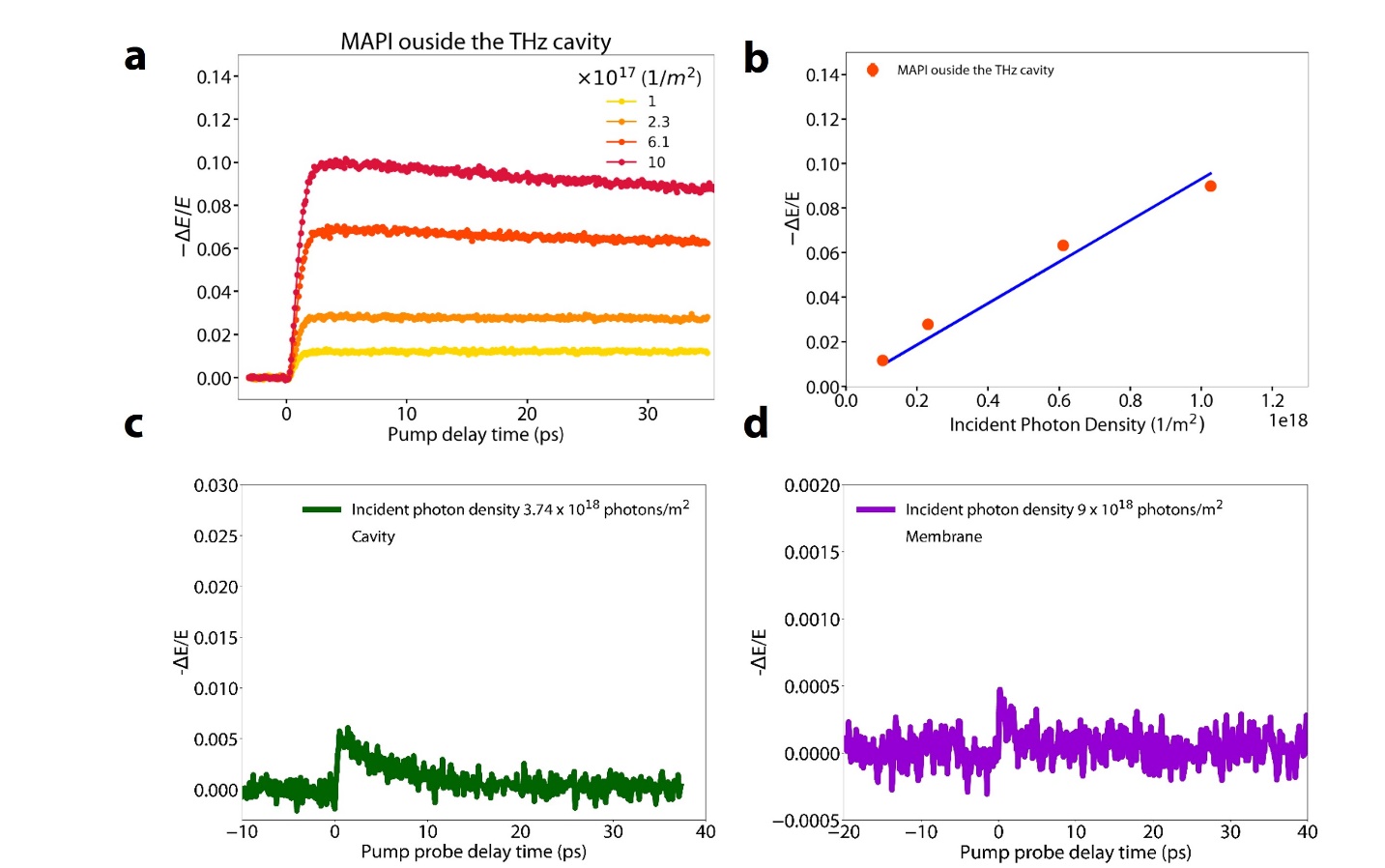


**Figure S4: Optical Pump THz Probe measurements. (a)** Photoinduced change of electric field ($-\Delta E/E$) at the maximum of THz pulse transmitted through MAPI outside of the THz cavity. The incident photon density is varied from $1\times{10}^{17}$ to $10\times{10}^{17}$photons m^-2^**. (b)** Values of $-\Delta E/E$ in panel (a) at 30 ps time delay for different incident photon densities. Fitted line (blue) shows that photoexciataion is in linear regime. **(c,d)** OPTP data (at maximum of THz pulse) for SiNx membrane and empty cavity with $x_{gap}=1 \text{cm}$, respectively.

**Supplementary Note 3**

**Simulation details**

Complex-valued refractive index of MAPI in ground and excited states is obtained using T-matrix formalism. To this end, calculated complex-valued transmission spectra are interatively fitted to experimental data (Figs. S5,6).


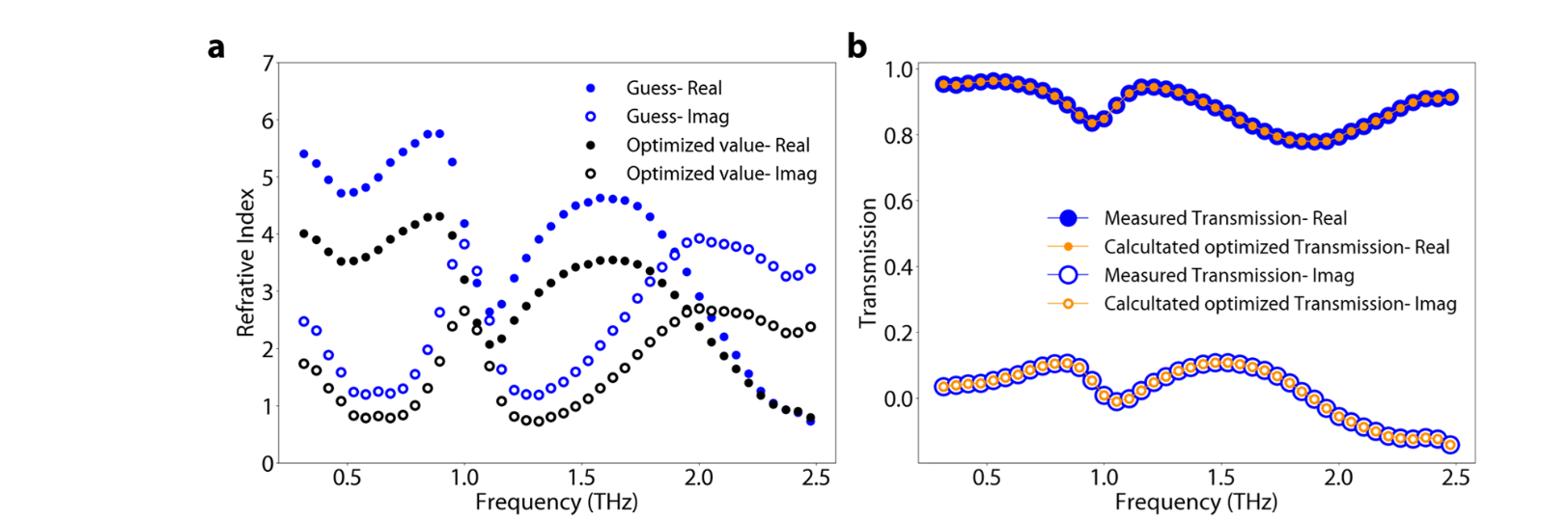


**Figure S5: Extraction of complex refractive index of MAPI in ground state. (a)** The guess values of refractive index are obtained from Tinkham approximation (blue). The optimized values of refractive index (black) are the result of the global minimization between calculated transmission and measured transmission. **(b)** Measured complex-valued transmission spectrum $\left( \frac{E_{sample}\left( \nu\right)}{E_{reference}\left( \nu\right)} \right)$ is shown in blue. $E_{reference}$ and $E_{sample}$ are THz fields transmitted through bare SiNx membrane and SiNx membrane coated with MAPI. Complex-valued transmission calculated using optimized refractive index of MAPI is shown in yellow.


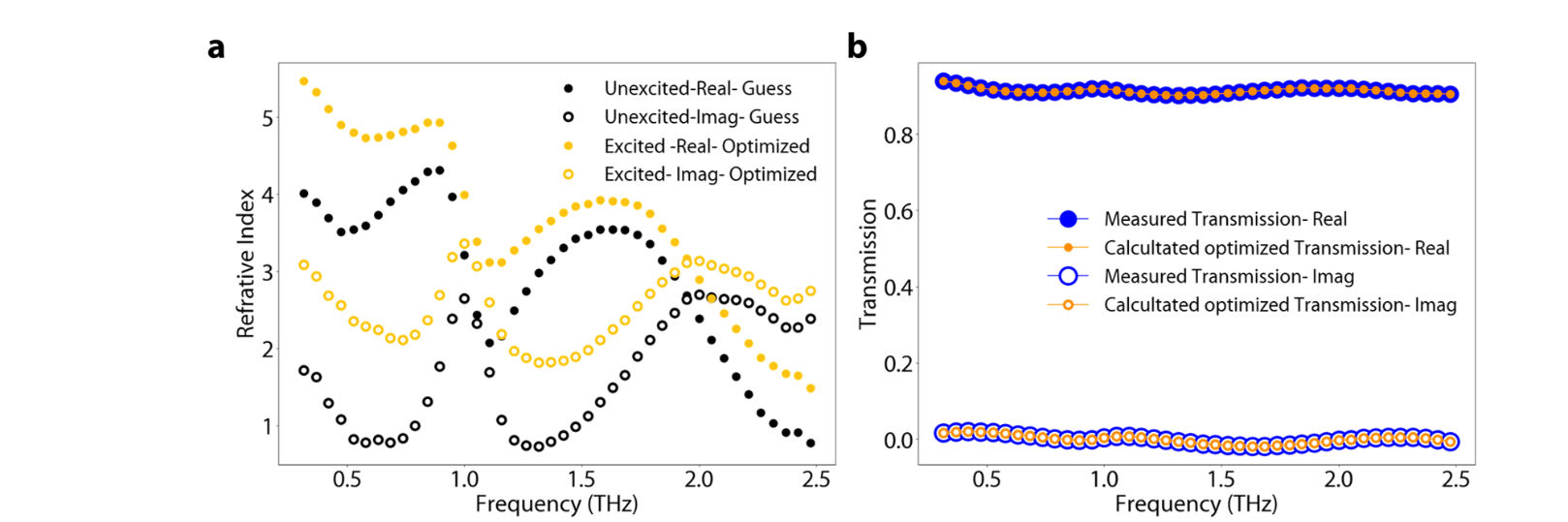


**Figure S6: Extraction of complex refractive index of MAPI in excited state. (a)** The guess values of refractive index are given by that of MAPI in ground state (black). The optimized values of refractive index (yellow) are the result of the global minimization between calculated transmission and measured transmission. **(b)** Measured complex-valued transmission spectrum $\left( \frac{E_{sample}\left( \nu\right)}{E_{reference}\left( \nu\right)} \right)$ is shown in blue. $E_{reference}$ and $E_{sample}$ are THz fields transmitted through SiNx membrane coated with MAPI in ground and excited states, respectively. Complex-valued transmission calculated using optimized refractive index of MAPI in the excited state is shown in yellow.

**
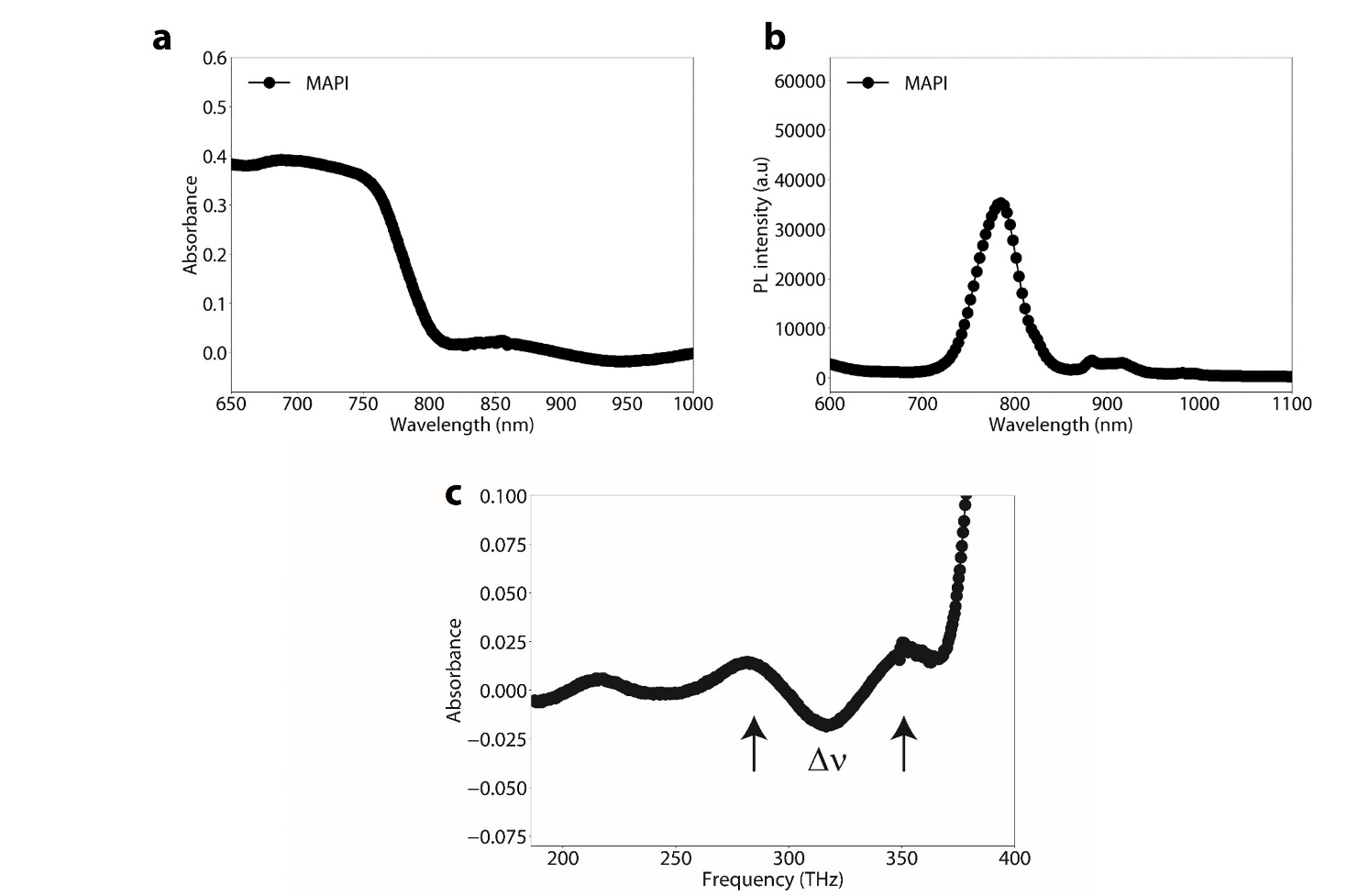
**

**Figure S7:** **Characterization of MAPI sample. (a,b)** Absorbance and photoluminescence intensity of MAPI deposited on SiNx membrane. **(c)** Data from (a) plotted against frequency. From the periodicity of the interference fringes ($\Delta\nu$) below the bandgap and using refractive index of n = 2.26^4^ we estimate MAPI film thickness around 1 μm.

**References**

1. Glover, R. E. & Tinkham, M. Transmission of Superconducting Films at Millimeter-Microwave and Far Infrared Frequencies. *Phys.Rev.* **104**, 844–845 (1956).

2. Naftaly, M. & Miles, R. E. Terahertz time-domain spectroscopy for material characterization. *Proc. IEEE* **95**, 1658–1665 (2007).

3. Krewer, K. L. *et al.* Accurate terahertz spectroscopy of supported thin films by precise substrate thickness correction. *Opt. Lett.* **43**, 447 (2018).

4. Löper, P. *et al.* Complex refractive index spectra of CH3NH3PbI3 perovskite thin films determined by spectroscopic ellipsometry and spectrophotometry. *J. Phys. Chem. Lett.* **6**, 66–71 (2015).
